# Lifetime estimation of thin-film transistors in organic emitting diode display panels with compensation

**DOI:** 10.1038/s41598-023-44684-5

**Published:** 2023-10-16

**Authors:** Jingyu Park, Sungju Choi, Changwook Kim, Hong Jae Shin, Yun Sik Jeong, Jong Uk Bae, Saeroonter Oh, Dae Hwan Kim

**Affiliations:** 1 School of Electrical Engineering, Kookmin University, Seoul, 02707 Republic of Korea; 2 Large Display Business Unit, LG Display Company, Paju, 10845 Republic of Korea; 3 Department of Electrical and Electronic Engineering, Hanyang University, Ansan, 15588 Republic of Korea

**Keywords:** Electrical and electronic engineering, Electronic devices

## Abstract

Oxide semiconductor thin-film transistors (TFTs) are used in the pixel array and gate driver circuits of organic light emitting diode (OLED) display panels. Long-term reliability characteristics of the TFTs are a barometer of the lifetime of OLED display panels. The long-term reliability of the driver TFTs is evaluated in a short time under high voltages and high temperature for an accelerated degradation test. If reliability parameters from the power law or stretched-exponential functions are the same for individual devices and devices in an operating panel, the lifetime of the panel can be accurately estimated. However, since compensation circuits are designed into operating panels, an environmental discrepancy exists between the accelerated test of single devices and the operation of devices in the panel. Herein, we propose a novel compensation stretched-exponential function (CSEF) model which captures the effect of the threshold voltage compensation circuit in the panel. The CSEF model not only bridges the discrepancy between individual devices and panel devices, but also provides a method to accurately and efficiently estimate the long-term lifetime of all display panels that utilize compensation circuits.

## Introduction

Organic light emitting diode (OLED) displays employ thin-film transistors (TFTs) in their active pixel array and in-panel gate driver circuits. Several types of pixel circuits exist depending on the compensation scheme and control signals, but basically all pixel circuits comprise a driver transistor and switching transistor. The driver TFT drives a current through the OLED to control the luminance of the pixel. The current is determined based on the gate-to-source voltage (*V*_*GS*_) and drain-to-source voltage (*V*_*DS*_) across the driver TFT, which is controlled using the data signal transferred by the switching TFT and voltage on the source node of the driver TFT. A turn-on voltage and data signal are applied to the gate and drain of the switching transistor, respectively, for a time duration of 1/*f*/*N*_*scan*_ for each frame time (= 1/*f*), where *f* is the refresh rate and *N*_*scan*_ is the number of pixels along the vertical direction of the display. For example, if the *f* = 120 Hz and *N*_*scan*_ = 2160 for an ultra-high definition television (UHD TV), signals to the switching transistor will be in the order of 3.8 μs every 8.3 ms. Pixel transistors are fabricated using oxide semiconductors or low-temperature polycrystalline silicon (LTPS). Oxide semiconductors exhibit good large-area uniformity up to generation 11 glass substrates (2940 mm × 3370 mm in size) making them suitable for large-screen displays in OLED TVs^1–3^. Moreover, oxide semiconductors also exhibit ultra-low off-state current (< 10^−15^ A) necessary for low-power energy-efficient displays in smartphones and smartwatches, where they are used in tandem with LTPS^4–7^. During operation of the display panel, the threshold voltage (*V*_*T*_) of the driver TFT shifts owing to electron trapping in the gate dielectric, and intrinsic defects in the channel material such as peroxy linkage or undercoordinated metal cations^8–13^. Any shift in the *V*_*T*_ (*ΔV*_*T*_) leads to brightness droop over time or brightness non-uniformity across the panel area. Therefore, ensuring satisfactory long-term reliability of TFTs is crucial with respect to the lifetime of OLED displays. Typically, TFTs are fabricated independently and subjected to harsh stress conditions (higher voltage, higher temperature) than normal operating conditions to accelerate degradation and assess reliability in a reasonable test time. This method is used as a proxy for assessing reliability of TFTs in a panel to estimate the panel lifetime during normal operation. Time dependence of *ΔV*_*T*_ of a single device is generally described using the power law, ${\Delta V}_{T}\left(t\right)\propto {t}^{b}$, or the stretched-exponential function (SEF),

1 $${\Delta V}_{T}\left(t\right)={\Delta V}_{T0}\left[1-{e}^{-{\left(t/\tau \right)}^{\beta }}\right]$$

which are used to depict the long-term reliability of the OLED panel^14,15^. If the parameters from *ΔV*_*T*_(*t*) of an individual device were the same as those of TFTs in a OLED panel, then this connection between individual TFTs and panel TFTs would not be an issue. However, in addition to the different voltage conditions between individual TFTs and TFTs in the panel, we discovered that the correlation begins to collapse when the devices and panel circuitry become increasingly more intricate. The most striking difference is that *V*_*T*_ compensation circuits are put in place for the driver TFTs in a panel environment^3,16,17^. Hence, the discrepancy between the accelerated stress measurement of an individual TFT and the long-term reliability of the panel is inevitable. Closing the gap between the reliability test of individual devices and panel devices is challenging; however, minimizing this discrepancy is important for ensuring accurate panel lifetime estimation. In this study, we identify this discrepancy and propose a compensated stretched-exponential function model that correctly captures the degradation of panel TFTs including the effect of the compensation circuit. By using this model, we can accurately estimate the long-term lifetime of display panels in a shorter time than conventional methods.

## Results

### Identification of inconsistency between individual device and panel device tests

The reliability of oxide semiconductor TFTs is a crucial factor that determines the lifetime of a display panel. The lifetime of display panels (e.g., commercial televisions) operated under normal usage should be 10 years or more. The failure of a display panel may occur due to long-term degradation of the OLED (due to aging and/or chemical damage) or the electronic devices controlling the OLEDs. The lifetime of the display panel is determined based on the failure of the OLED or TFT, whichever occurs earlier. This study focuses on the lifetime of the oxide semiconductor TFT backplane. The critical lifetime of the TFT is realized when the worst-case *V*_*T*_ reaches *V*_*T*_(*τ*_*life*_) = *V*_*T*_(*t* = 0) + *ΔV*_*T,limit*_, where *ΔV*_*T,limit*_ is a predetermined value set depending on the upper limit the compensation circuit can effectively operate and accurately compensate the *ΔV*_*T*_. Owing to the slow degradation process, accelerating the degradation by testing the devices under high voltages and temperatures in a matter of hours is a common practice. However, the correlation between the accelerated reliability test of a single TFT device and the lifetime of an OLED panel fails when the panel and devices become increasingly complex. Hence, the panel lifetimes are often extrapolated from electrical measurements of panel degradation over months-long test operation. Finding the reason behind this discrepancy and validating a consistent and quantitative correlation between device and panel lifetime is crucial in significantly shortening the panel evaluation time and accurately representing the TFT array in a panel environment.

**Figure 1:**
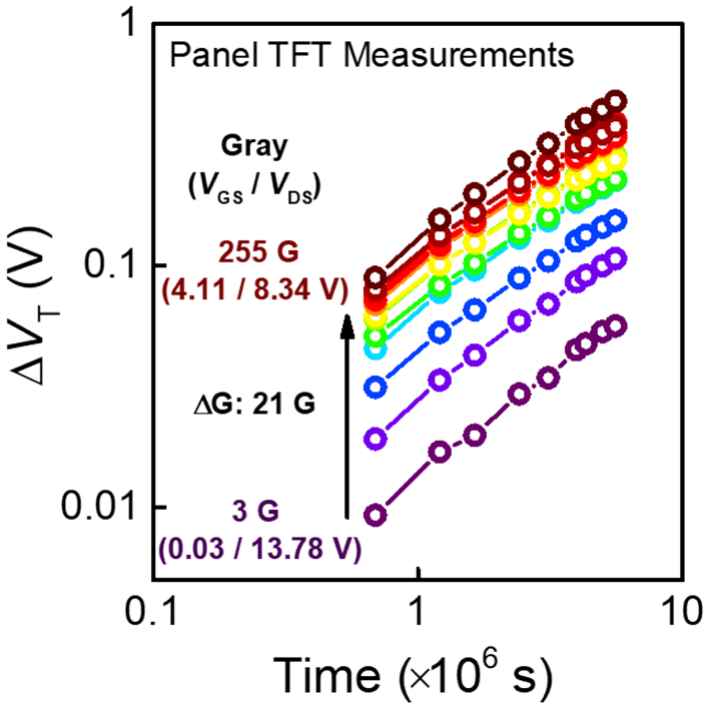
*V*_*T*_ shift of transistors in the display panel subject to different gray levels varying from 3 to 255 G, up to 1500 h in a room temperature ambient. Each gray level is determined based on the gate overdrive voltage (*V*_*GS*_ – *V*_*T*_) and drain-to-source voltage (*V*_*DS*_) applied to the driving transistor, represented in the figure as the first and second numbers in parenthesis, respectively. Difference in gray levels between curves is 21 G apart.

Figure 1 shows the *ΔV*_*T*_ with respect to TFTs in an operating display panel under different gray levels up to 1500 h at room temperature. The gray level is defined as the digitized brightness level of a pixel, with 0 G being the darkest and 255 G being the brightest level. Gray levels are expressed based on calibrated voltage conditions including gate overdrive voltage *V*_*OV*_ (= *V*_*GS*_ – *V*_*T*_) and drain-to-source voltage *V*_*DS*_ conditions applied to the driver TFTs. The *ΔV*_*T*_ is higher for higher gray levels because the TFTs are subject to high bias stress conditions. Hence, examining whether the device reliability tests correlate to the reliability of TFTs in the pixel array of a panel is extremely important.

**Figure 2:**
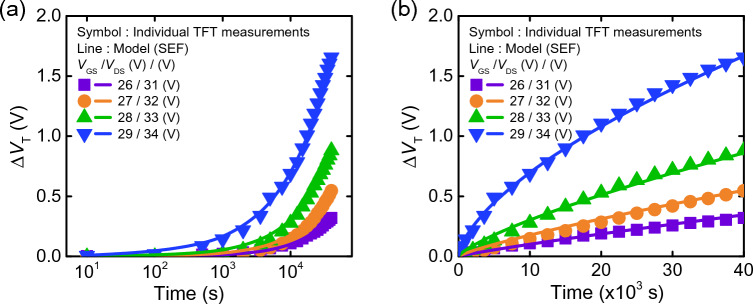
*V*_*T*_ shift of individual transistors for different *V*_*GS*_ and *V*_*DS*_ combinations, with time *x*-axis in (**a**) log scale, and (**b**) linear scale. Measurements are represented using symbols and stretched-exponential function (SEF) model values are represented using solid lines.

Figure 2 shows the *ΔV*_*T*_ of an InGaZnO (IGZO) TFT device under DC stress using different gate-to-source voltage (*V*_*GS*_) and drain-to-source voltage (*V*_*DS*_) conditions. Symbols in Fig. 2 represent the measured data and the lines represent SEF fitting curves. Table 1 shows the SEF parameters (*ΔV*_*T0*_, *τ*, *β*) for different bias stress conditions. Generally, the extracted *τ* and *β* values differ upon how *ΔV*_*T0*_ is defined. *ΔV*_*T0*_ is the saturated value of *ΔV*_*T*_ when the TFT has been subject to stress for a sufficiently long time (*t* → ∞).

**Table 1:** Stretched-exponential function model parameters of an individual IGZO TFT device under DC stress of different bias conditions.

*ΔV*_*T0*_ = *V*_*GS*_ – *V*_*T*_	20 V	21 V	22 V	23 V	24 V	25 V	26 V	27 V	28 V	29 V
*τ* (s)	1.5 × 10^7^	1.2 × 10^7^	9.7 × 10^6^	9.8 × 10^6^	8.7 × 10^6^	1.0 × 10^7^	8.7 × 10^6^	8.2 × 10^6^	7.6 × 10^6^	7.3 × 10^6^
*β*	0.90	0.80	0.85	0.90	0.80	0.85	0.90	0.85	0.80	0.75

**Figure 3:**
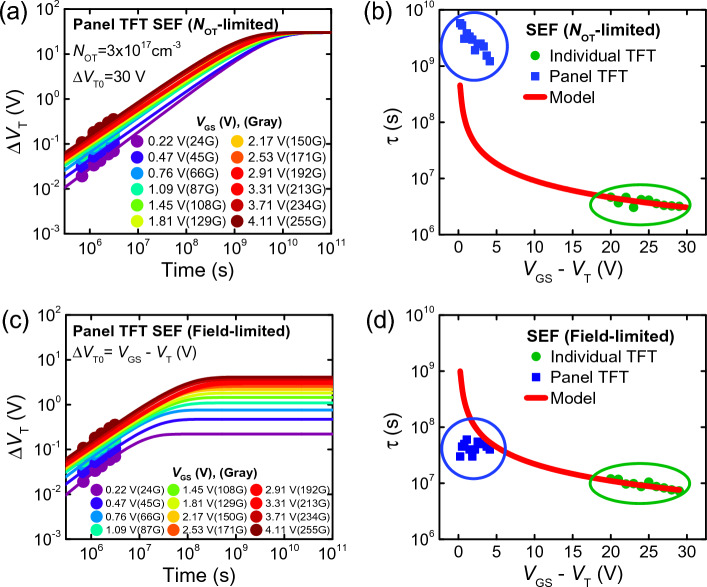
(**a**, **c**) Threshold shift (*ΔV*_*T*_) of IGZO TFTs in the display panel subject to various bias conditions corresponding to a range of gray levels. Symbols represent measurement values up to 1500 h, while solid lines represent modeling results extended to 10^11^ s using the (**a**) *N*_*OT*_-limited model and the (**c**) electric-field-limited model. (**b**, **d**) Characteristic time constant *τ* from reliability tests of individual devices (green squares) and devices in a panel (blue circles). *τ* values are extracted from SEF using the (**b**) *N*_*OT*_-limited model and (**d**) electric-field-limited model. A calculated inverse relationship between *τ* and *V*_*OV*_ is represented by the solid red line. Panel *τ* values deviate from the inverse relationship under both models.

When the *N*_*OT*_*-limited model* is used, we assume that the cause of *ΔV*_*T*_ saturation over time is caused by the finite trap density in the interface and bulk of the gate dielectric (*N*_*OT*_). In this model, *ΔV*_*T0*_ is a constant that is irrelevant to the stress voltage and is determined by *N*_*OT*_ present in the device. *ΔV*_*T0*_ is determined based on the maximum *ΔV*_*T*_ caused by *N*_*OT*_ being completely full of trapped charge.

2 $${\Delta V}_{T0}=\frac{q{N}_{OT}\cdot {t}_{ox}}{{C}_{ox}}$$

where *q* is the elementary charge, *C*_*ox*_ is the gate oxide capacitance per area, and *t*_*ox*_ is the thickness of the gate dielectric layer. Figure 3a shows the *ΔV*_*T*_(*t*) of the panel TFTs for different gray levels, where *N*_*OT*_ = 3 × 10^17^ cm^−3^ is assumed which gives *ΔV*_*T0*_ = 30 V. All *ΔV*_*T*_(*t*) curves saturate at the same *ΔV*_*T0*_ value, where hypothetically all the defect states in the GI are filled with trapped electrons at *t* → ∞. The characteristic time *τ* in the SEF (Eq. 1) can be extracted using the measured *ΔV*_*T*_(*t*) for panel and single individual devices (Eq. 2). Also, *τ* follows an inverse relationship with (*V*_*OV*_ = *V*_*GS*_ – *V*_*T*_) as expressed in the following equation:

3 $$\uptau ={\left[{\sigma }_{0}\cdot {v}_{th}\cdot {f}_{MB}\cdot {n}_{s}\right]}^{-1}={\left[{\sigma }_{0}\cdot {v}_{th}\cdot {f}_{MB}\cdot {C}_{ox}\cdot \left({V}_{GS}-{V}_{T}\right)/q\cdot {t}_{act}\right]}^{-1}$$

where *σ*_*0*_ is the capture cross section, *v*_*th*_ is the thermal velocity, *f*_*MB*_ is the Maxwell–Boltzmann distribution function, *n*_*s*_ is the carrier volume density, and *t*_*act*_ is the thickness of the active channel layer. The calculated *τ* (Eq. 3) and the *τ* extracted from panel and individual device measurements using the *N*_*OT*_-limited model are plotted against (*V*_*GS*_ – *V*_*T*_) in Fig. 3b. We discovered that the *τ* extracted from stress tests of single individual devices agree with the calculations; however, the *τ* extracted from panel TFTs do not follow the calculated *τ*-*V*_*OV*_ trend, exhibiting noticeably higher values. Hence, the *N*_*OT*_-limited SEF model is not appropriate for predicting the lifetime of TFTs in a display panel.

In the *electric-field-limited model*, the *ΔV*_*T*_ saturates due to the weakening of the electric field across the gate insulator. This is because the *V*_*T*_ shifts positively under stress owing to the trapped charge while *V*_*GS*_ is constant, hence decreasing *V*_*GS*_ – *V*_*T*_(*t*) over time. In the electric-field-limited model, we define

4 $${\Delta V}_{T0}={V}_{GS}-{V}_{T}\left(t=0\right),$$

5 $${\Delta V}_{T}\left(t\right)=\left[{V}_{GS}-{V}_{T}(t=0)\right]\cdot \left[1-{e}^{-{\left(\frac{t}{\tau }\right)}^{\beta }}\right].$$

Hence, *ΔV*_*T0*_ is dependent on the stress *V*_*GS*_ and initial *V*_*T*_ of the device. Figure 3c shows the *ΔV*_*T*_(*t*) of the panel TFTs for different gray levels using the electric-field-limited model, where devices subject to low *V*_*GS*_ – *V*_*T*_(*t* = 0) saturates at low *ΔV*_*T0*_ values. Figure 3d plots the extracted and calculated characteristic time *τ* against *V*_*OV*_ using the electric-field-limited SEF model. The *τ* extracted from *ΔV*_*T*_(*t*) of individual devices under reliability tests follow the inverse correlation with *V*_*OV*_. However, *τ* extracted from panel TFTs do not follow this correlation with the calculated values and exhibit lower values. The field-limited SEF model does not capture the *ΔV*_*T*_(*t*) for panel TFTs and has weak correlation with that of individual TFTs, and hence cannot be used as a prediction model for the panel lifetime. Therefore, both SEF models do not accurately represent the *V*_*T*_(*t*) of TFTs in a panel environment, raising the question of whether self-consistency between individual TFT devices and panel TFTs can be achieved. Therefore, if a model could be developed wherein the *τ* of panel TFTs follows the inverse (*V*_*GS*_ – *V*_*T*_) relation, we could gain confidence that lifetime models between single devices and devices in the panel are consistent. Then, why does the evaluation of TFTs in the panel result in *τ* values that do not follow the inverse *V*_*OV*_ relation? Herein, we propose a novel model to bridge this discrepancy.

### Proposed compensated stretched-exponential function model

Primary difference in evaluation conditions between a reliability test for an individual device and devices in the panel is whether the bias conditions are maintained constant or not. Constant voltage is maintained throughout the stress phase of an instability test of a single individual device. However, OLED display panels have a compensation circuit that compensates the varying *V*_*T*_ in real-time, indicating that the bias conditions change dynamically as the *V*_*T*_ of devices in the pixel array shift. Hence, we must first determine whether the SEF fitting is fundamentally appropriate for devices in a panel with dynamic *V*_*T*_ compensation.

When the *V*_*T*_ shifts due to stress or a prolonged period of normal operation, *V*_*T*_ can be represented as *V*_*T*_ = *V*_*T*_(*t* = 0) + *ΔV*_*T*_(*t*), where *V*_*T*_(*t* = 0) is the initial *V*_*T*_ and *ΔV*_*T*_(*t*) is the time-dependent *V*_*T*_ shift. For *V*_*GS*_ – *V*_*T*_ to be kept constant, the compensation circuit applies *V*_*GS*_ + *ΔV*_*T*_(*t*) instead of *V*_*GS*_ to the driver TFT. Thus, the SEF can be modified as follows:

6 $${\Delta V}_{T}\left(t\right)=\left[\left({V}_{GS}+{\Delta V}_{T}\left(t\right)\right)-{V}_{T}(t=0)\right]\left[1-{e}^{-{\left(t/\tau \right)}^{\beta }}\right]$$

from Eqs. 1 and 4, assuming the electric-field-limited case. The *ΔV*_*T*_(*t*) term is present on both sides of Eq. 6; hence, by rearranging the terms we obtain the *compensated stretched-exponential function* (CSEF), expressed as follows:

7 $${\Delta V}_{T}\left(t\right)=\left[{V}_{GS}-{V}_{T}(t=0)\right]\cdot \left[\frac{1}{{e}^{-{\left(\frac{t}{\tau }\right)}^{\beta }}}-1\right].$$

**Figure 4:**
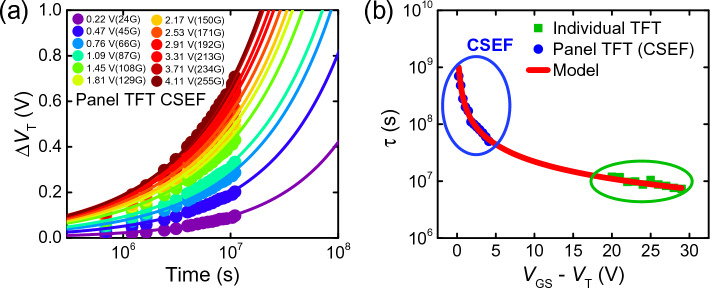
(**a**) *ΔV*_*T*_ of IGZO TFTs in the display panel subject to various bias conditions corresponding to a range of gray levels. Symbols are measured values up to 3,600 h, while the solid lines are fitting curves using the compensated stretched-exponential function (CSEF) model. (**b**) *τ* values extracted using the CSEF model for TFTs in a panel with compensation (blue circles) and the SEF model for individual TFTs (green squares). All extracted values follow the Inverse relationship between *τ* and *V*_*OV*_ (red solid line).

CSEF can be used to extract the *τ* and *β* from devices in an active panel with *V*_*T*_ compensation. Figure 4a shows the CSEF model fitting to *V*_*T*_ shifts of devices in a panel for various gray levels up to 3600 h at room temperature. First, we implement the CSEF model to devices on the display panel with a constant brightness represented by a gray level ranging from 24 to 255 G. The measured *V*_*T*_(*t*) of TFTs in the panel with respect to various constant gray levels are overlaid on top of CSEF model curves with parameters *τ* and *β* corresponding to each gray level in Fig. 4a. Table 2 lists the CSEF parameters for various brightness levels. The CSEF parameter *τ* values from the panel TFTs are plotted against *V*_*GS*_ –* V*_*T*_ juxtaposed with those of individual TFT devices on the same plot in Fig. 4b. The modified *τ* extracted from panel devices now follows the inverse relation with (*V*_*GS*_ – *V*_*T*_), justifying the hypothesis that indeed the compensation was the cause of the discrepancy between panel devices and individual devices. The validity of the CSEF model is proven, having *τ* extracted from both single individual devices and panel devices being in agreement on a universal *τ*–*V*_*ov*_ curve (Eq. 3). By selecting a SEF model based on the stress/operation concept of the device under test, we can maintain consistency between data obtained from standalone devices and devices within a panel. For TFT devices with no compensation the standard SEF model is used, while for TFT devices in a panel with compensation circuit operation the CSEF model is used.

**Table 2:** Parameters for panel TFTs at various bias conditions each corresponding to a brightness (in gray levels) using the proposed CSEF model.

Brightness	025 G	045 G	066 G	087 G	108 G	127 G	150 G	171 G	192 G	213 G	234 G	255 G
*ΔV*_*T0*_ (V)	0.22	0.47	0.76	1.09	1.45	1.81	2.17	2.53	2.91	3.31	3.71	4.11
*τ* (s)	2.5 × 10^8^	1.7 × 10^8^	1.2 × 10^8^	9.7 × 10^7^	7.8 × 10^7^	6.5 × 10^7^	5.5 × 10^7^	4.8 × 10^7^	4.2 × 10^7^	3.8 × 10^7^	3.4 × 10^7^	3.1 × 10^7^
*β*	0.63	0.62	0.68	0.68	0.72	0.62	0.73	0.82	0.78	0.78	0.77	0.82

### Verification of the CSEF model via various display data patterns

**Figure 5:**
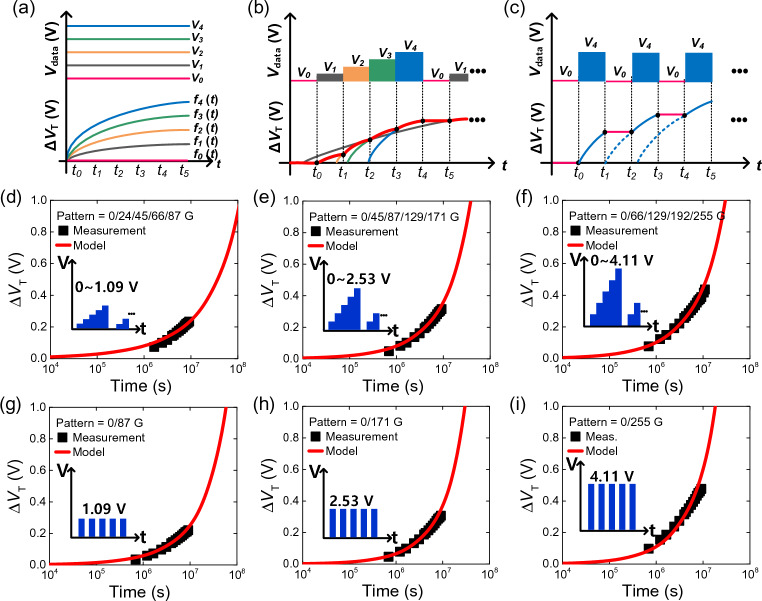
(**a**–**c**) Methodology for modeling the *ΔV*_*T*_(*t*) of TFTs in a display panel subject to arbitrary patterns, such as (**b**) cycling of 5 gray levels, and (**c**) on and off cycling pattern with 50% duty. Estimation of *ΔV*_*T*_ of panel TFTs under a 5–gray pattern (measurement values in square shapes, CSEF model in solid lines) for various gray level combinations: (**d**) 0, 24, 45, 66, and 87 G (*V*_*GS*_ = 0–1.09 V); (**e**) 0, 45, 87, 129, and 171 G (*V*_*GS*_ = 0–2.53 V); and (**f**) 0, 66, 129, 192, and 255 G (*V*_*GS*_ = 0–4.11 V). Estimation of *ΔV*_*T*_ of panel TFTs using the CSEF model (solid line) in comparison to measured values (square shapes) for an ON/OFF pattern cycling between 0 G (*V*_*GS*_ = 0 V) and (**g**) 87 G (*V*_*GS*_ = 1.09 V), (**h**) 171 G (*V*_*GS*_ = 2.53 V), and (**i**) 255 G (*V*_*GS*_ = 4.11 V), respectively.

Having established the CSEF model, it is necessary to verify its validity for TFTs in the panel for various data patterns. To emulate a display operation environment rather than a constant DC stress bias, we select rolling patterns between 5 gray levels and an on and off pattern with a duty cycle of 50%. To model the *ΔV*_*T*_(*t*) for TFTs in the panel subject to different patterns, a systematic approach must be adopted. Figure 5a–c show how the *ΔV*_*T*_(*t*) can be obtained for an arbitrary pattern by adjoining *ΔV*_*T*_(*t*) segments each with a constant gray level for a certain duration. Each pattern segment corresponds to a gray level that is correlated to data bias conditions (*V*_*GS,i*_, *V*_*DS,i*_) for a particular pattern duration (*Δt*_*i*_). The *ΔV*_*T*_(*t*) during an arbitrary pattern segment can be expressed as:

8 $$F\left( {t{-}t_{i} } \right) = f_{i} \left( {t_{i,x} + \Delta t_{i} } \right) + f_{i + 1} \left( {t{-}t_{i + 1,x} } \right), t_{i} = t_{i - 1} + \Delta t_{i} , t_{i + 1,x} \;{\text{is}}\;{\text{where}}\;f_{i + 1} \left( {t_{i + 1,x} } \right) = f_{i} \left( {t_{i,x} + \Delta t_{i} } \right)$$

where *F*(*x*) represents the *ΔV*_*T*_(*t*) from the panel pattern, and *f*_*i*_(*t*) represents the individual Δ*V*_*T*_(*t*) for a specific gray level condition corresponding to the voltage pulse *V*_*GS,i*_ between *t*_*i-1*_ to *t*_*i*_ (*Δt*_*i*_ = *t*_*i*_ – *t*_*i-1*_). Each pattern segment uses one set of CSEF parameters corresponding to the constant gray levels, as listed in Table 2. The degradation history of *V*_*T*_ caused by the data pattern up to that current point is reflected, by keeping the final Δ*V*_*T*_ value of the previous segment as the initial value of the current segment. Furthermore, since *ΔV*_*T*_(*t*) is updated and not reset after each segment, the change in degradation rate is also considered. For example, the first few segments can be expressed as:

9 $$F\left( {t{-}t_{0} } \right) = f_{1} \left( {t{-}0} \right)$$

10 $$F\left( {t{-}t_{1} } \right) = f_{1} \left( {\Delta t_{1} } \right) + f_{2} \left( {t{-}t_{2,x} } \right),\,t_{1} = t_{0} + \Delta t_{1} ,\,t_{2,x} \;{\text{is}}\;{\text{where}}\;f_{2} \left( {t_{2,x} } \right) = f_{1} \left( {\Delta t_{1} } \right)$$

11 $$F\left( {t{-}t_{2} } \right) = f_{2} \left( {t_{2,x} + \Delta t_{2} } \right) + f_{3} \left( {t{-}t_{3,x} } \right),t_{2} = t_{1} + \Delta t_{2} ,\,t_{3,x} \;{\text{is}}\;{\text{where}}\;f_{3} \left( {t_{3,x} } \right) = f_{2} \left( {t_{2,x} + \Delta t_{2} } \right)$$

and so on. By using this method, we can replicate the *ΔV*_*T*_ of panel TFTs experiencing complicated data patterns. Figure 5d–f show the application of the CSEF model using Eq. 8 for a rolling pattern of 5 gray levels for 3600 h using three different gray level combinations. Figure 5g–i show the application of the proposed method on a panel under an alternating on and off pattern with a duty cycle of 50%. We can observe that the *ΔV*_*T*_(*t*) measurements and model results are in good agreement, validating the panel data pattern-dependent CSEF model. We can accurately predict the panel lifetime under arbitrary complex display patterns using CSEF model parameters obtained from measurement data of panel TFTs under various gray level conditions.

### CSEF model with V_T0_ variation using empirical V_T0_-dependent τ model

**Figure 6:**
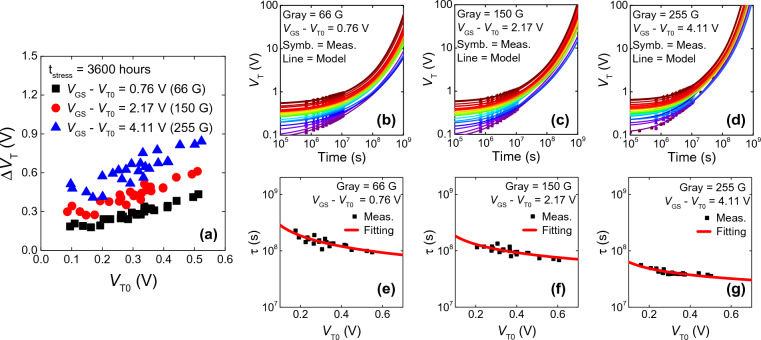
(**a**) Distribution of *ΔV*_*T*_ of 24 devices with *V*_*T0*_ variation plotted against *V*_*T0*_. A positive correlation is consistently observed across different gray levels. *V*_*T*_(*t*) variation of 24 panel devices operated under selected gray levels: (**b**) 66 G, (**c**) 150 G, (**d**) 255 G. Measurement values are denoted by shapes, and CSEF model curves are shown in solid lines. Extracted *τ* values from CSEF plotted against *V*_*T0*_ of the devices: (**e**) 66 G, (**f**) 150 G, (**g**) 255 G. Fitted empirical functions of *τ* are shown in red solid lines.

Owing to the amorphous nature of oxide semiconductors, it is inevitable but to have a statistical variation in device properties across the large glass substrate spanning over 3 m on one side. Based on the verified panel data-dependent CSEF model, we analyze the initial *V*_*T*_ (= *V*_*T0*_) distribution in the CSEF model, specifically by establishing a relation between *V*_*T0*_ and *τ*. Figure 6a shows the *ΔV*_*T*_ of 24 panel devices with variation plotted against *V*_*T0*_. The devices are operated at a constant gray level for 3600 h. Despite maintaining a constant *V*_*OV*_ throughout the operation with the help of the compensation circuit, a positive correlation between *ΔV*_*T*_ and *V*_*T0*_ can be observed. This shows that devices with high *V*_*T0*_ will most likely result in high *ΔV*_*T*_. Figure 6b–d show the *ΔV*_*T*_(*t*) of 24 devices in the display panel at a constant brightness level (only few selected gray levels are shown).

In Eq. 3, any change in *V*_*T0*_ does not affect *n*_*s*_ because compensation corrects any *ΔV*_*T*_(*t*) and variation in *V*_*T0*_ to ensure that *V*_*OV*_ is kept constant. Even under the same intended fabrication process, the local spatial variation in process conditions influence the film properties and the device characteristics, causing variation in *V*_*T0*_. Process factors that vary *V*_*T0*_ could also influence the trap properties. Particularly, we assume *V*_*T0*_ affects the capture cross-section *σ*, and an effective *V*_*T0*_-dependent *τ*(*V*_*T0*_) is introduced. When we use the *V*_*T0*_-dependent *τ*(*V*_*T0*_) in the CSEF model as in:

12 $${\Delta V}_{T}\left(t\right)=\left[{V}_{GS}-{V}_{T0}\right]\cdot \left[{exp\left(-{\left(\frac{t}{\tau \left({V}_{T0}\right)}\right)}^{\beta }\right)}^{-1}-1\right],$$

the model captures the *ΔV*_*T*_(*t*) including *V*_*T0*_ variation, which agrees with measured values with respect to different gray levels, as shown in Fig. 6b–d. Average *τ* and *β* values obtained from the CSEF model of 24 devices are listed in Table 3. To find the functional form of *τ*(*V*_*T0*_), variation in *τ* values are plotted against *V*_*T0*_ as shown in Fig. 6e–g. We implement an empirical equation in the form of $\uptau ={a}_{0}\cdot {{V}_{T0}}^{{b}_{0}}$, where *a*_*0*_ and *b*_*0*_ are used as fitting parameters. These fitting parameters for each brightness level are listed in Table 3. For a higher *V*_*T0*_, *τ* becomes smaller because *b*_*0*_ < 0 (and *a*_*0*_ > 0) which leads to *ΔV*_*T*_(*t*) increasing rapidly at an earlier timescale. *a*_*0*_ is smaller and *b*_*0*_ has a less negative value for a higher *ΔV*_*T0*_ which corresponds to a smaller *τ* value that is less sensitive to* V*_*T0*_ at a higher gray level. Our proposed CSEF model can be useful in estimating the lifetimes of display panels including the effect of *V*_*T0*_ variation of the TFT devices.

**Table 3:** Average of CSEF parameters across 24 TFTs with statistical variation for various brightness levels. Fitting parameters *a*_*0*_ and *b*_*0*_ for the empirical functional form of $\uptau ={a}_{0}\cdot {{V}_{T0}}^{{b}_{0}}$.

Brightness	025 G	045 G	066 G	087 G	108 G	127 G	150 G	171 G	192 G	213 G	234 G	255 G
*ΔV*_*T0*_ (V)	0.22	0.47	0.76	1.09	1.45	1.81	2.17	2.53	2.91	3.31	3.71	4.11
Average *τ* (s)	1.2 × 10^8^	1.0 × 10^8^	1.0 × 10^8^	8.5 × 10^7^	6.8 × 10^7^	6.5 × 10^7^	5.2 × 10^7^	4.2 × 10^7^	3.8 × 10^7^	3.3 × 10^7^	3.1 × 10^7^	2.8 × 10^7^
Average *β*	0.67	0.63	0.68	0.76	0.73	0.56	0.74	0.82	0.80	0.75	0.72	0.83
*a*_*0*_ (s V^−b^)	7.1 × 10^7^	6.4 × 10^7^	6.8 × 10^7^	5.9 × 10^7^	5.9 × 10^7^	5.8 × 10^7^	5.9 × 10^7^	5.2 × 10^7^	4.7 × 10^7^	3.7 × 10^7^	2.7 × 10^7^	2.7 × 10^7^
*b*_*0*_	− 0.98	− 0.91	− 0.63	− 0.66	− 0.57	− 0.51	− 0.49	− 0.42	− 0.53	− 0.33	− 0.40	− 0.38

## Discussion

**Figure 7:**
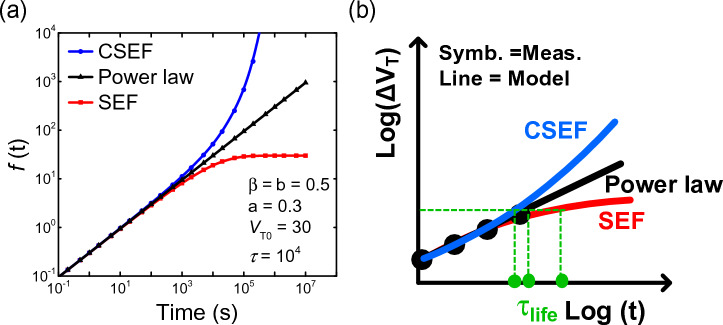
(**a**) Comparison of the functional form between the power law, SEF, and CSEF model. Exponents of time are set equal (*β* = *b* = 0.5). (**b**) Extraction of the panel lifetime using the CSEF model, power law, and SEF.

Conventionally when evaluating the lifetime of the panel, the power law or SEF are used to model *ΔV*_*T*_(*t*) of TFTs. We have demonstrated that the CSEF model represents driver TFTs more accurately under a *V*_*T*_ compensation scheme compared to the SEF model. Herein, we quantitatively compare the functional form of the power law (${\Delta V}_{T}\left(t\right)=a\cdot {t}^{b}$), SEF (Eq. 1), and CSEF (Eq. 7). Figure 7a shows the three models as a function of time. Since *b* and *β* determine the slope of the functions in a log–log plot, we equate them to be the same value, *b* = *β*. We note that when *t* $\ll$ *τ*, the functions approximately overlap. When *t* $\ll$ *τ* and 0 < *β* < 1 are satisfied, the SEF can be approximated by the power law, as in asymptotic power laws^18,19^. The power law is not based on physical parameters; it is a mathematical approximation of the SEF. Because the power law has a simple form, it is often widely employed in the industry instead of SEF. Conversely, when time becomes comparable to or larger than *τ*, the curves differ from one another in the following order: *ΔV*_*T,CSEF*_ > *ΔV*_*T,powerlaw*_ > *ΔV*_*T,SEF*_, where SEF saturates at *ΔV*_*T*_*,*_*SEF*_(*t → *∞) = *ΔV*_*T0*_. For TFTs in display panels, we observe that the CSEF describes and reproduces the panel measurement data, and CSEF parameters follow the same *V*_*OV*_^−1^ relation as followed by the SEF parameters of individual TFTs. As shown in Fig. 7b, when the panel lifetime is short, the selection among the three models may have less importance. However, as a longer product lifetime is desired, and the TFTs and circuit schemes become increasingly sophisticated, stable, and robust, the SEF or power law underestimates the *ΔV*_*T*_ when extrapolated beyond measurement data, thus posing the risk of overestimating the lifetime of the display panel.

The stress conditions for reliability evaluation with respect to individual TFTs and panel TFTs are different in voltage and temperature levels, as well as operating methods. Hence, a discrepancy is observed in *ΔV*_*T*_ progression. Moreover, owing to the absence of an appropriate model, panels are subject to long testing times of up to thousands of hours. We have proposed a novel CSEF model that captures the *ΔV*_*T*_(*t*) of TFTs in a panel with *V*_*T*_ compensation circuits. The proposed CSEF model is verified by comparing the measurement data of *ΔV*_*T*_(*t*) up to 3600 h. Additionally, the efficacy of the proposed model is proven by accurately estimating the *ΔV*_*T*_(*t*) of TFTs in a panel with respect to different data patterns. We can shorten the panel lifetime evaluation time to the amount of time required to extract CSEF parameters from panel TFTs at various gray levels, estimate the lifetime of panels under various arbitrary display rolling patterns, and extend the range of lifetime estimation far beyond conventional extrapolation methods. Furthermore, the CSEF model does not overestimate the panel lifetime as opposed to the widely-used power law or the standard SEF. Thus, using the CSEF model for estimation would be more rigorous, especially for panels with long lifetimes.

## Methods

Self-aligned top-gate structure amorphous IGZO TFTs are fabricated herein^10^. First, a bottom metal layer is deposited on the glass substrate to serve as a light shield, followed by the deposition of a SiO_2_ buffer layer via plasma-enhanced chemical vapor deposition (PECVD). The semiconductor active layer is formed via DC sputtering of a-IGZO (In:Ga:Zn = 1:1:1), followed by gate stack formation comprising a SiO_2_ gate insulator formed by PECVD, and a Cu gate formed via sputtering. During patterning of the gate stack, the dry etch and plasma treatment forms the highly conductive source/drain access regions. Interlayer dielectric is deposited by the PECVD of SiO_2_, and contact holes are formed for Cu S/D electrodes to fill via sputtering. The source node of the driver TFT is electrically connected to the light shield layer. A SiO_2_ passivation layer is deposited by PECVD. Width and length of the devices are 18 μm and 8.5 μm, respectively. Electrical properties are measured using a semiconductor parameter analyzer (4156C, Agilent). *V*_*T*_ of the individual TFTs are extracted where *I*_*D*_ = 5 nA from the saturation current characteristics at *V*_*DS*_ = 10 V. Stress conditions for the reliability tests of the standalone individual TFTs are *V*_*GS*_ = 20–30 V and *V*_*DS*_ = *V*_*GS*_ + 5 V to ensure saturation operation at room temperature. Stress time is 4 × 10^4^ s and recovery time is 10^4^ s.

For the panel evaluation, *V*_*T*_(*t*) of the driver TFT in the pixel is recorded for 3600 h, for constant brightness of various gray levels and different test data patterns. *V*_*T*_ of the driver TFT is obtained from a read-out circuit in the panel. The change in *V*_*T*_ is compensated during operation using an external compensation circuit^2^. The panel region is divided into different sections, wherein each section of the panel is subject to either a constant gray level or rolling test patterns. For constant gray levels, the brightness of the white color is varied from 0 to 255 G, with increments of 21 G. For rolling test data patterns, an ON/OFF pattern and a rolling 5-level pattern are used.

CSEF parameters are extracted from the measurement data of panel TFTs by using:

13 $$\mathrm{ln}\left[\mathrm{ln}(1+\frac{{\Delta V}_{T}\left(t\right)}{{\Delta V}_{T0}})\right]=\beta \left(\mathrm{ln}\left(t\right)-\mathrm{ln}\left(\tau \right)\right),$$

which is a rearrangement of Eq. 7. When the left-hand side of Eq. 13 is plotted against ln(*t*), *τ* and *β* can be found from the *x*-axis intercept and slope, respectively.

## Data Availability

The data that support the findings of this study are available from the corresponding author upon reasonable request.

## References

[1] Shin H-J (2015). Novel OLED display technologies for large-size UHD OLED TVs. SID Symp. Dig. Tech. Pap..

[2] Shin H-J (2021). A novel 88-inch 8K OLED display for premium large-size TVs. SID Symp. Dig. Tech. Pap..

[3] Wu Z (2022). Development of ultra-large 95 inch 8 K 120 Hz OLED display. SID Symp. Dig. Tech. Pap..

[4] Kato K (2012). Evaluation of off-state current characteristics of transistor using oxide semiconductor material, indium–gallium–zinc oxide. Jpn. J. Appl. Phys..

[5] Saito N (2019). High mobility (>30 cm^2^ V^−1^ s^−1^) and low source/drain parasitic resistance In–Zn–O BEOL transistor with ultralow <10−20 A *μ*m^−1^ off-state leakage current. Jpn. J. Appl. Phys..

[6] Chang T-K, Lin C-W, Chang S (2019). LTPO TFT technology for AMOLEDs. SID Symp. Dig. Tech. Pap..

[7] Chung U-J (2020). Manufacturing technology of LTPO TFT. SID Symp. Dig. Tech. Pap..

[8] Jang JT (2022). Cation composition-dependent device performance and positive bias instability of self-aligned oxide semiconductor thin-film transistors: Including oxygen and hydrogen effect. ACS Appl. Mater. Interfaces.

[9] Choi S (2022). Excessive oxygen peroxide model-based analysis of positive-bias-stress and negative-bias-illumination-stress instabilities in self-aligned top-gate coplanar In–Ga–Zn–O thin-film transistors. Adv. Electron. Mater..

[10] Park J (2023). Current boosting of self-aligned top-gate amorphous InGaZnO thin-film transistors under driving conditions. Adv. Electron. Mater..

[11] Choi S (2017). Systematic decomposition of the positive bias stress instability in self-aligned coplanar InGaZnO thin-film transistors. IEEE Electron Device Lett..

[12] De Meux ADJ, Poutois G, Genoe J, Heremans P (2018). Defects in amorphous semiconductors: The case of amorphous indium gallium zinc oxide. Phys. Rev. Appl..

[13] Lopes ME (2009). Gate-bias stress in amorphous oxide semiconductors thin-film transistors. Appl. Phys. Lett..

[14] Ji Z, Lin L, Zhang JF, Kaczer B, Groeseneken G (2010). NBTI lifetime prediction and kinetics at operation bias based on ultrafast pulse measurement. IEEE Trans. Electron Devices.

[15] Lee J-M, Cho I-T, Lee J-H, Kwon H-I (2008). Bias-stress-induced stretched-exponential time dependence of threshold voltage shift in InGaZnO thin film transistors. Appl. Phys. Lett..

[16] Shin H-J (2018). A novel OLED display panel with high-reliability integrated gate driver circuit using IGZO TFTs for large-sized UHD TVs. SID Symp. Dig. Tech. Pap..

[17] Choi S (2020). Positive bias stress instability of InGaZnO TFTs with self-aligned top-gate structure in the threshold-voltage compensated pixel. IEEE Electron Device Lett..

[18] Metzler R, Klafter J (2002). From stretched exponential to inverse power-law: Fractional dynamics, Cole–Cole relaxation processes, and beyond. J. Non-Cryst. Solids.

[19] Powell MJ, van Berkel C, Hughes JR (1989). Time and temperature dependence of instability mechanisms in amorphous silicon thin-film transistors. Appl. Phys. Lett..

